# Difficulties at Burton

**Published:** 1921-11

**Authors:** 


### DIFFICULTIES AT BURTON.

(FROM A CORRESPONDENT.)

The General Committee of the Burton-on-Trent Infirmary has passed the following resolution: "That the Infirmary Committee are of opinion that it is desirable, in the best interest of the hospital, to continue as a voluntary institution, and to remain outside the scheme proposed by the Voluntary Hospitals Commission."

From a somewhat condensed report, which should therefore be received with caution, it appears that this resolution was addressed to the General Purposes Committee of the Burton Borough Council, which had been requested by the Secretary of the Voluntary Hospitals Commission to help in forming a local Voluntary Hospitals Committee. This letter explained that the Commission had decided that the best course would be to establish one Committee for the county and county boroughs of Burton-on-Trent, Smethwick, Stoke-on-Trent, Walsall, West Bromwich, and Wolverhampton, and that the Commission was anxious for the co-operation of the Borough Council in the selection of the local Committee.

After discussing the two letters from the hospitals and from the Commission, the General Purposes Committee of the Council unanimously resolved that, "having regard to the circumstances existing in Burton-upon-Trent," no useful purpose would be served by the entry of the county borough into the scheme, and that the Town Clerk should so inform the Secretary of the Commission and the Clerk of the Staffordshire County Council.

The face value of these decisions suggests a very regrettable state of affairs, which, however, one cannot but think may be amended by further consideration. If the resolution of the Committee of the Burton-on-Trent Infirmary be studied carefully it will be seen that the reason for its decision to stand aside from the scheme proposed by the Voluntary Hospitals Commission is a laudable desire "to continue as a voluntary institution." In other words, the assumption behind the resolution is that the scheme of the Hospitals Commission, if not the Commission itself, is inimical to the voluntary principle. But this surely is a mistaken, if not a grotesque, assumption. It is impossible to read and digest the report of the Cave Commission, out of which the Voluntary Hospitals Commission was begotten, without seeing that the aim of both is to strengthen and preserve the voluntary system. Why, then, does this suspicion exist? We suppose the reason to be that the Commission is to distribute the Government grant of half a million, and that this grant is suspected of being the thin end of the wedge. The sum is relatively so small and the number of voluntary hospitals is so large that its influence towards State control is negligible. Indeed, it was granted for fear lest the collapse of the voluntary hospitals should force the State to take them over. There is, we believe, no desire, and no means, for such a colossal scheme, least of all in the minds of its supposed authors. Perhaps the resolution of the Infirmary merely means that it expects nothing from the grant, or that it is not in need of it. If either is the explanation, then the misunderstanding is worse. For the object of the Hospitals Commission and of the local Committees through which it will largely work is not only to distribute the Government grant, but to use that grant to secure attention for recommendations toward the better grouping and more co-ordinate working of the voluntary hospitals in each area. Every principle has its weakness when it comes to be applied, and the weakness of the voluntary principle of hospital support has been a want of co-ordination between hospitals. There has been too much isolation and too little mutual aid, too much overlapping and too much waste of beds and energy, too much competition and too little co-operation. Since, as we believe and as the report of the Cave Committee insisted, the voluntary principle must be maintained, it is safe as well as wise to remedy this weakness. Any hospital which rejects the new scheme and refuses to participate in it may be doing great harm to the very principle which it professes to support so zealously.

It remains to be seen what the Hospitals Commission will do next. It has ample arguments of the kind suggested here. Has it more than arguments, more than persuasive powers?

